# Cloud-based blockchain technology to identify counterfeits

**DOI:** 10.1186/s13677-022-00341-2

**Published:** 2022-10-20

**Authors:** Vinodhini Mani, M. Prakash, Wen Cheng Lai

**Affiliations:** 1 School of Computing, Department of Computer Science and Engineering, SRM Institute of Science and Technology, Kattankulathur - 603 203, Chennai, Tamil Nadu India; 2 School of Computing, Data Science and Business System, SRM Institute of Science and Technology, Kattankulathur - 603 203, Chennai, Tamil Nadu India; 3 Bachelor Program in Industrial Projects, National Yunlin University of Science and Technology, Douliu, 640301 Taiwan; 4 Department of Electronic Engineering, National Yunlin University of Science and Technology, Douliu, 640301 Taiwan

**Keywords:** Privacy, Distributed ledger, Supply chain visibility, Access control, Pharmaceutical supply chain

## Abstract

Multi-stakeholder and organizational involvement is an integral part of the medicine supply chain. Keeping track of the activities associated with medical products is difficult when the system is complex. Their complexity limits transparency and data provenance. Deficiencies within existing supply chains result in the counterfeiting of drugs, illegal imports, and inefficient operations. Due to these limitations, product integrity is compromised, resulting in product wastage. Visibility of the entire product supply chain is crucial for the pharmaceutical industry in terms of product safety and reduction of manufacturing costs. The Cloud-based Blockchain-powered architecture of the system provides a platform for addressing the need of pharma-material traceability, data storage, privacy of data, and quality assurance. This framework comprises of the identification of activities through tagging, information sharing in a secure environment; cloud-based storage using an off-chain Interplanetary File System (IPFS) and an on-chain couch DB; and access to this information that is controlled by the system's regulator. Electronic drug records will be accessed via a smart contract in Hyperledger Blockchain. The system assists in identifying false and cross-border products through the manufacturer and country of origin. A scan will identify counterfeit medications, showing that they are unauthorized products which may pose a risk to patients. Our experiments demonstrated the efficiency and usability of the design platform. Finally, we benchmarked the system using Hyperledger Caliper.

## Introduction

Distribution points in the supply chain deliver pharmaceutical products to consumers in bulk. Drugs are transferred between parties during the supply chain life cycle— from manufacturers to intermediaries, pharmacy chains, hospitals, logistics companies, and the end users (patients). Pharma-manufacturers are responsible for manufacturing products according to certain specifications, and ensuring the correct packaging and labelling of the items with relevant information. Distribution companies package and transport pharmaceutical products to pharmacies that retail the products. Pharmacies, clinics, and hospitals across the city will be available for customers to purchase drugs. Several parties are involved in the supply chain of medical products, including manufacturers, suppliers, wholesalers, supermarkets, pharmacies, and patients.

More diseases emerge every day, leading to the introduction of new drugs with different names in the market. In the United States, about $200 billion is lost each year in business as a result of drug counterfeiting [1, 2]. Many children are killed by fake drugs in developing countries, according to the World Health Organization (WHO) [3, 4]. Blockchain [5] can help reduce operational costs, and improve transparency and traceability. Business and consumers are able to see more of the supply chain with greater transparency. For high-value goods such as diamonds and pharmaceutical drugs, blockchain can improve the supply chain transparency and reduce fraud. Blockchain delivers scalability, security, disaster recovery, and reliability to enterprises.

### Motivation

**Fig. 1:**
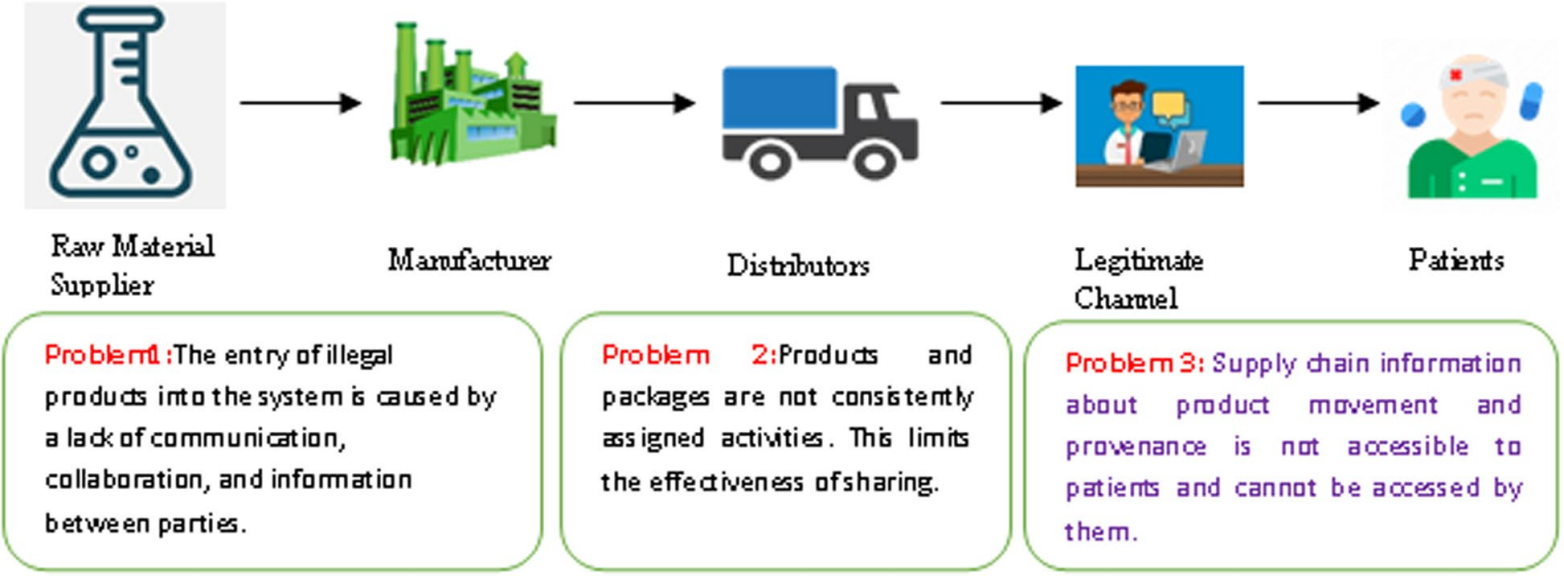
A Challenge of Collaboration

Every healthcare system strives to deliver medicine on time to patients. Pharmacists are considered elegant and dignified when it comes to managing their supply chain. Pharmacies, shipping companies, retailers, and end users are all stakeholders in this process. A product's supply chain activity cannot be tracked. Information about the product is not available to the patient (Fig. 1).

### Contribution

Pharma-manufacturers can track the movement of their products throughout the supply chain with this blockchain-based solution. With it, patients can verify exactly where their medication comes from, and where it was manufactured with an instant QR code. The major contributions of this research are as follows: The proposed pharmaceutical supply chain network takes into account possible stakeholders, such as the drug producer, the shipper, the retailer, and the customer.

**Fig. 2:**
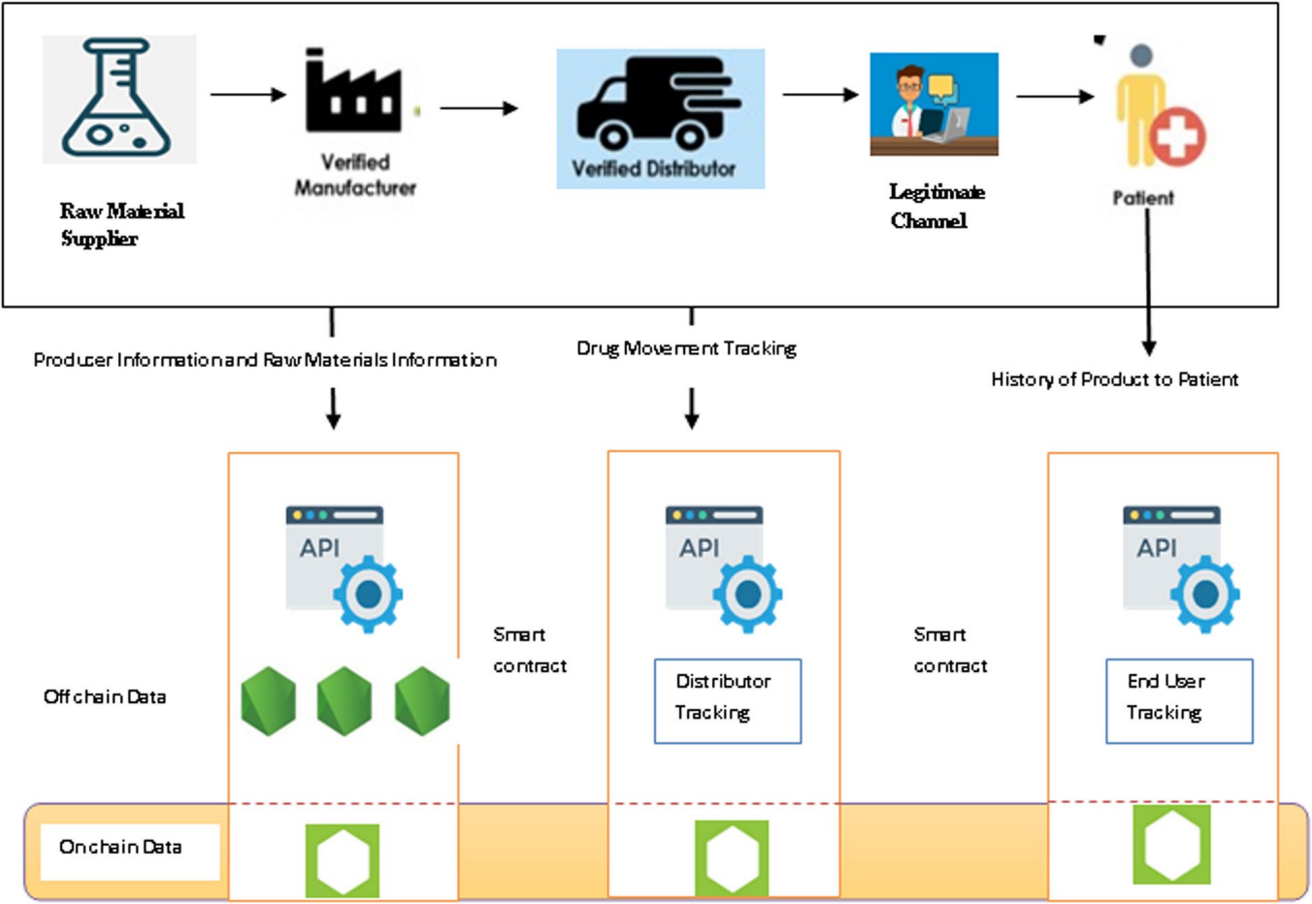
Contributions of the Research

- Users can be classified as administrator, regulator, producer, shipper, retailer, or customer. The classification is generated when they register in the application.
- Each node is hosted and managed by the participant, and the data remains in the participant's own environment.
- The node verifies every product movement with smart contracts and encrypts all the data before it is loaded.
- By participating in the network, participants have control over what information is written to and seen in the ledger.
- Users can query ledger information across nodes using smart contracts and APIs rather than physically transferring the data.
- Fig. 2 shows the contribution of the paper.

The remainder of this paper is organized as follows: "Related works" section analyses existing blockchain-based drug delivery solutions. The design of smart contracts and the methodology for the proposed system are described in "Blockchain-enabled pharma supply chain" section. "Implementation of pharma supply chain system" section describes how the proposed system implements smart contracts, models with distributed ledger storage, and smart contract modelling. Results are presented in "Performance evaluation" section. The paper is concluded in "Conclusion" section.

## Related works

A traceable medication can be verified as legitimate, and can reflect the flow of transactions executed between stakeholders so that they can verify its composition and origin [6]. To improve transparency in the supply chain, Ashok Kumar Pundir et al. in [7] discussed areas of affliction. Considering the recent instances of counterfeits in the country, transparency and accountability are important features in any supply chain. A growing number of stakeholders and the number of products being manufactured have resulted in the complexity and sophistication of supply chain networks. According to [8], blockchain can be used for various applications, including tracking and tracing. It can also deliver greater efficiency if the processes are optimized and automated.

The authors in [9] developed a new supply chain management system based on blockchain. According to their research, many actors and processes are involved in the supply chain. These authentication components allow a client or client application access to the blockchain and the relevant data in it. They concluded that overcoming cultural barriers and technological obstacles are crucial, but that energy efficiency will give us an advantage in the environmental and economic arena. A manufacturer's supply chain information is one of the most important tools when creating a competitive supply chain, according to [10]. As a result of multiple code schemes, the supply chain in a globalized environment finds it difficult to share information.

In [11], the authors worked on a blockchain solution with the same goal of enabling secure information sharing and increasing transparency, which has the potential to save the industry hundreds of millions of dollars. [12] proposed a blockchain solution for pharma supply chain management that satisfies privacy, security, and control transparency in all transactions. However, this system lacks scalability and is not suitable for large supply chain networks. The current literature supports the development of novel decentralized, transparent, and secure solutions that can combat the counterfeiting of drugs [13–15]. However, it lacks transparency in issues such as product data, which should be open to all consumers.

Models that use blockchain for data storage makes the system more scalable, and provides privacy [16]. Patient-centric data storage and sharing is implemented using chaincode based on blockchain technology in [17, 18]. [19] discussed the challenges in the supply chain. He stated that the framework for the supply chain should include privacy, access control visibility, and secure storage and sharing. Supply chain networks are improving with blockchain technology in many blockchain startups [20, 21]. The authors of [22, 23] conducted research on COVID-19 patient health analysis using blockchain technology. The security-based patient-centric healthcare was developed in [24–26]. It implements blockchain technology with fog computing to concentrate on the security of the system. The healthcare industry needs to develop technologically-enhanced solutions and frameworks that can provide security, authentication, access control, end-to-end visibility, and identity systems for stakeholders.

### Importance of blockchain in the pharma Industry

Pharmaceutical companies use blockchain technology in a variety of ways. Serialization has addressed the security threat of falsified medication. Data miners are deployed to maintain quality control from the manufacturer to the pharmacy. This is done through digital signatures, blockchain chain codes, health information, and digital signature [27].

### Blockchain technology for the prevention of counterfeit drugs

Pharmaceutical products are serialized and assigned security features that can be verified by consumers and differentiated from counterfeits. The blockchain system also enhances security through transparent and chaincode-based transactions. Providing consumers with high-quality and safe drugs requires trust and transparency in the pharmaceutical industry. Lack of trust can lead to counterfeiting, which poses a safety risk to the public. Using blockchain, drug safety can be enhanced, and counterfeit drugs can be detected, leading to improved safety and a decreased mortality rate. It is possible to use IPFS and the Ethereum blockchain to implement a variety of approaches. Blockchain has been implemented for small firms, and was analyzed in [28].

## Blockchain-enabled pharma supply chain

**Fig. 3:**
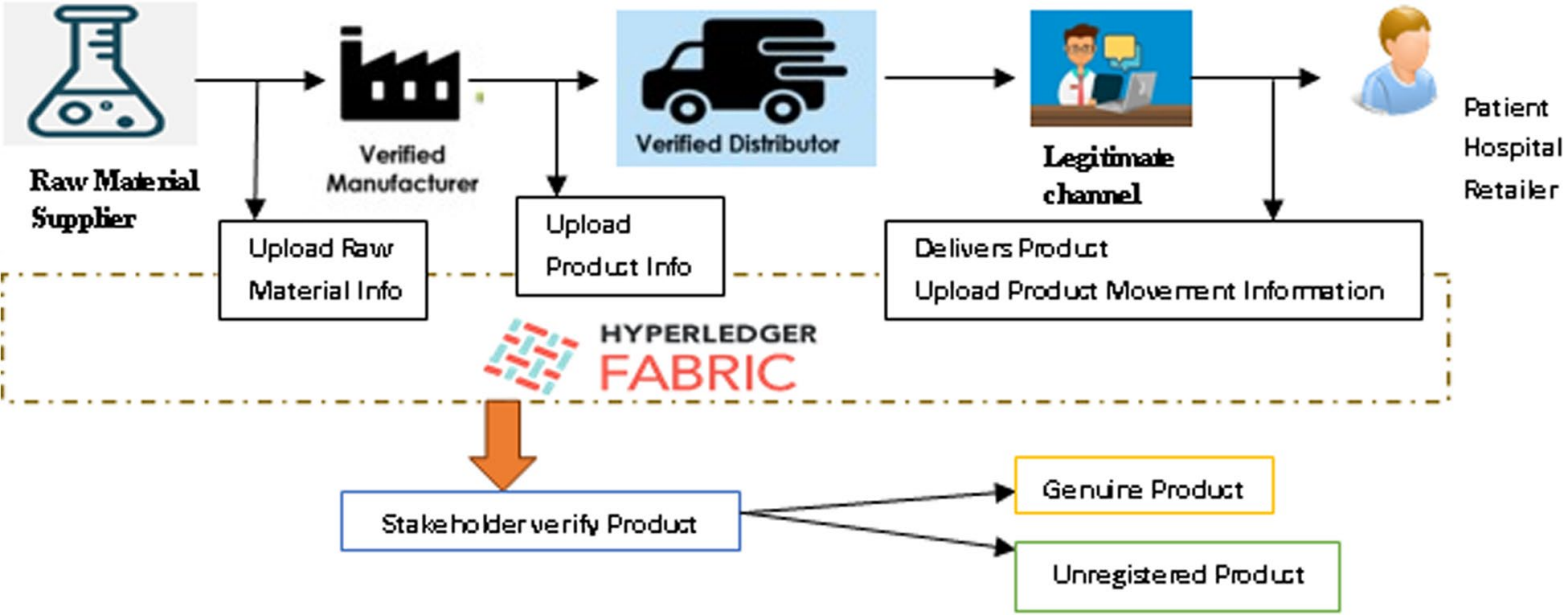
The Proposed Framework

The proposed framework as shown in Fig. 3 will allow stakeholders to track the movement of products through the supply chain. Hyperledger fabric provides a secure and safe service for viewing the history of the drug supply chain. Our fabric utilizes the Byzantine-fault tolerant consensus to ensure safe and reliable communication in an untrusted environment [16, 19]. The components of Hyperledger fabric are certificate authority, peer nodes and ordering service. Certificate authority generates unique certificates for every stakeholder in the network. The transaction process is handled by peers in the blockchain. Transaction blocks are ordered through services before they are committed to the network. Our Hyperledger fabric provides privacy, efficient processing of transactions, and chaincode facilities to write access control rules using access control language. Over 3,500 transactions can be executed per second through the Hyperledger fabric [20]. This system guides the consumer through the enlistment, shipping, and purchasing process. With complete visibility of the supply chain, customers can track their orders from start to finish. The application shows the order history, shipping history, and receipt history of a retailer and the customer who logged into the application. The regulator who oversees the proper implementation of the procedures has access to all orders in the system.

### Proposed frameworks

Using this system, pharmaceutical manufacturers can accelerate global safety while reducing costs in the supply chain by monitoring and ensuring the security of the process. This system was designed to handle:

- (i) Identification of activities through tagging.
- (ii) Information sharing in a secure environment and cloud-based storage using an off-chain Interplanetary File System (IPFS) and on-chain couch DB.
- (iii) Access to this information that is controlled by the system's regulator.

#### Identification of activities through tagging

Each product, package, or unit must be both physically labelled and digitally tagged with an individual encrypted code. Integrated warehouse activities with client orders and government serials would be identified utilizing certificate authority digital ID software.

#### Information sharing in a secure environment and cloud-based storage using an off-chain Interplanetary File System (IPFS) and on-chain couch DB

The system ensures data accuracy and protects data ownership by utilizing a non-intrusive method of information exchange. All parties agree to provide every batch and product with complete information about their activities.

#### Access to this information that is controlled by the system’s regulator

The source of a product should be checked immediately to ensure it is genuine. Cloud-based IPFS off-chain and Hyperledger fabric on-chain provide a safe and secure environment for information. It helps to identify fake and cross-border products using blockchain technology. Counterfeit medication is clearly visible following the scanning process, as it signals that it is an illegal product and may pose a risk to patients. When unregistered pharmaceuticals are detected, an alert is sent to the stakeholders such as the distributors, pharmacists, and patients who purchase the medical products. If the unregistered pharmaceuticals are investigated, there will be no further crime. Regulation bodies are notified to act immediately in case of counterfeit medicine sales.

### System architecture

**Fig. 4:**
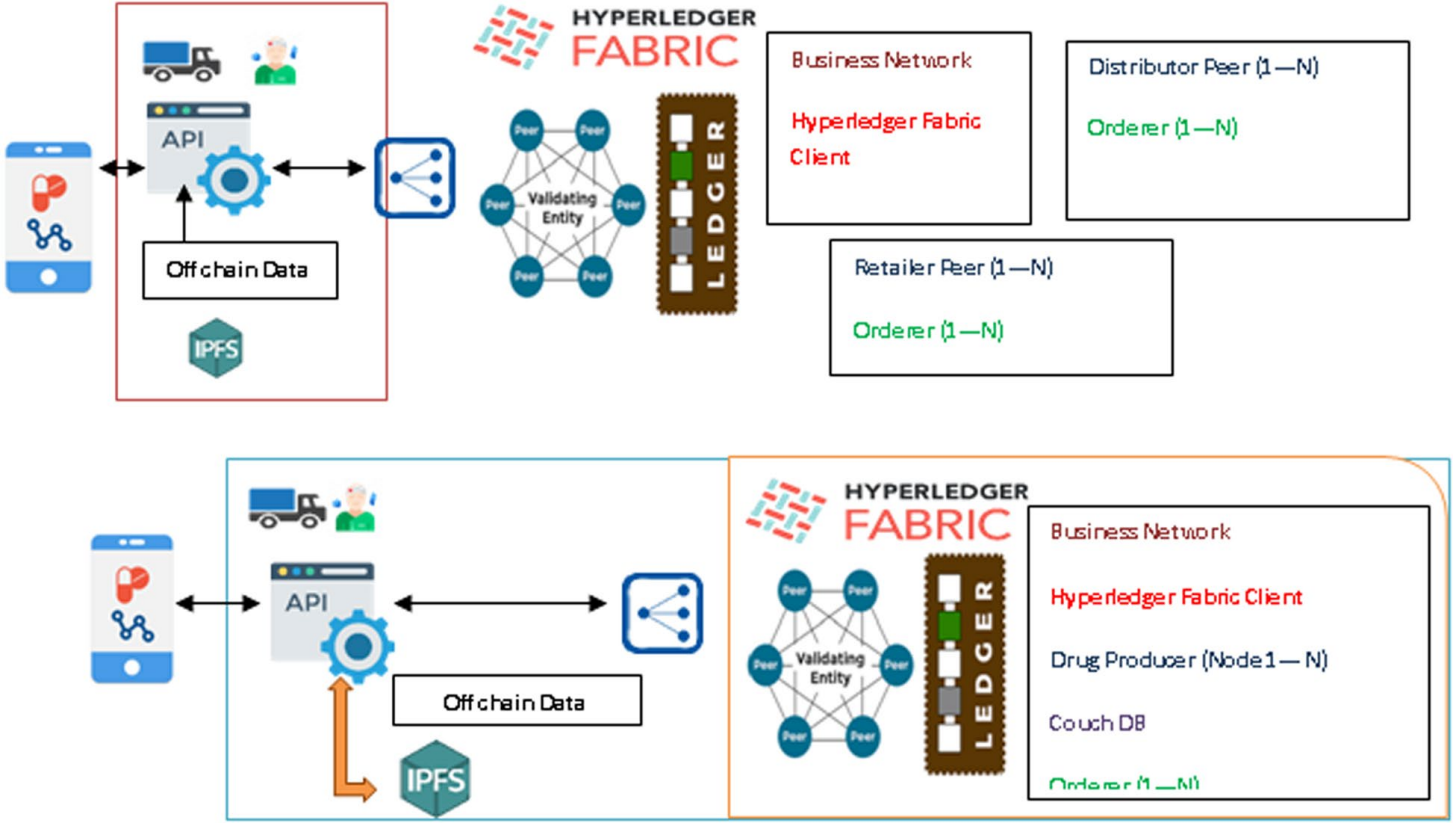
The System Architecture

As seen in the proposed architecture shown in Fig. 4, Hyperledger fabric provides highly reliable, scalable, and flexible blockchain-based distributed ledger solutions. To accommodate the complexity and intricacies of the economic ecosystem, it is designed so that different components can be plugged in. An API has been developed and can be installed for tracking the product by stakeholders. The product information will be databased on an off-chain system called the Cloud-based IPFS after encryption. The hash value of the encrypted product is stored in the Onchain Hyperledger database. To host a Blockchain network, the Hyperledger Fabric Framework contains several components including a ledger, membership identity provider, smart contracts, peers, ordering services, channels, certification authorities, and organizations. State databases, otherwise known as state ledgers, are used in conjunction with blockchains to form ledgers. Due to the immutability of ledgers, the blockchain allows only appending operations. Members have access credentials to participate in transactions conducted over the Hyperledger Fabric network via the membership identity service. Blockchain is based on smart contracts. As part of the network, peers are responsible for maintaining ledgers and transactions. This serves as the Onchain to store the hash value of encrypted data. They order transactions based on a first-come-first-serve basis for all channels. Ordering services are independent of peer processes, and order transactions into a block independently from peer processes. Channels provide data isolation and confidentiality over a public blockchain. Transacting parties have to be authenticated for channels to interact with them, and a channel-specific ledger is shared between peers in the channel. Network members and their users receive certificates based on the public key infrastructure from the Certificate Authority. Blockchain service providers invite companies and organizations to join the network. A membership service provider adds an organization to the network.

### Fabric deployment interaction

**Fig. 5:**
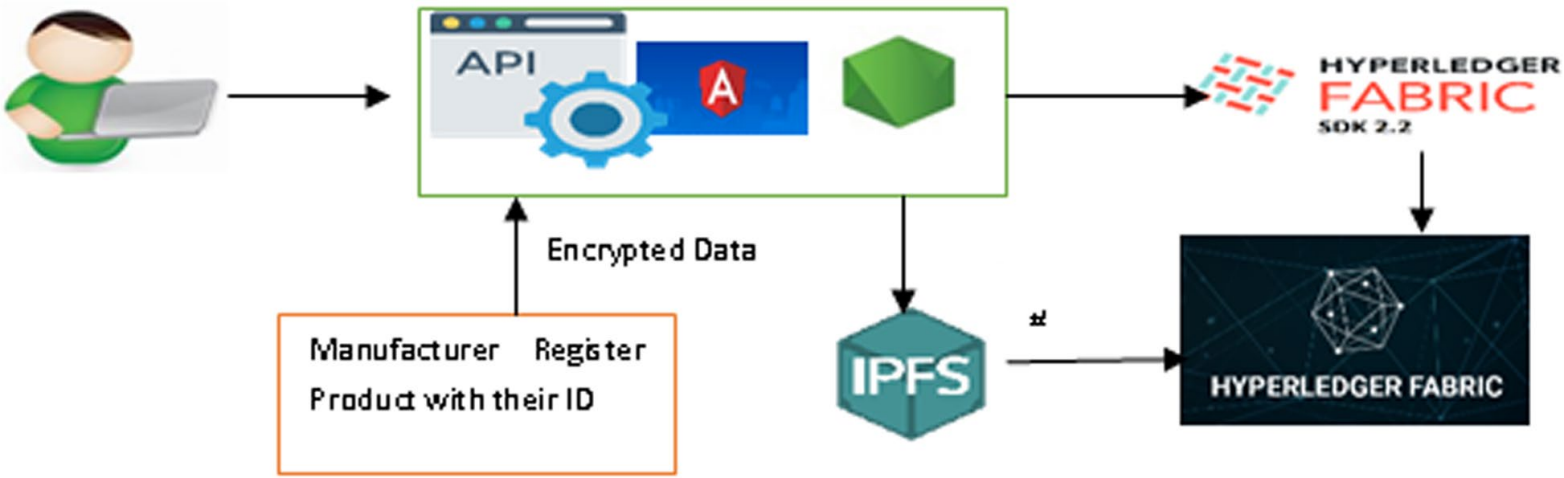
Data Flow from User to Application

The component interaction is shown in Fig. 5. An Angular Web UI allows the user to interact with the blockchain and Cloud-based IPFS, and query its state and ledger. An application API of the Node.js backend server is used by the UI. Application servers use Fabric SDK APIs to interact with the library. In addition to interaction with and submission of transactions to a deployed Hyperledger Fabric 1.4.1 network, the SDK can also receive events from the network. Using Hyperledger fabric, any aspect of this network can easily be customized, including node locations, the hardware CPU and memory, endorsement policies, and new members and organizations that need to be integrated.

### Pharma supply chain— transaction workflow

**Fig. 6:**
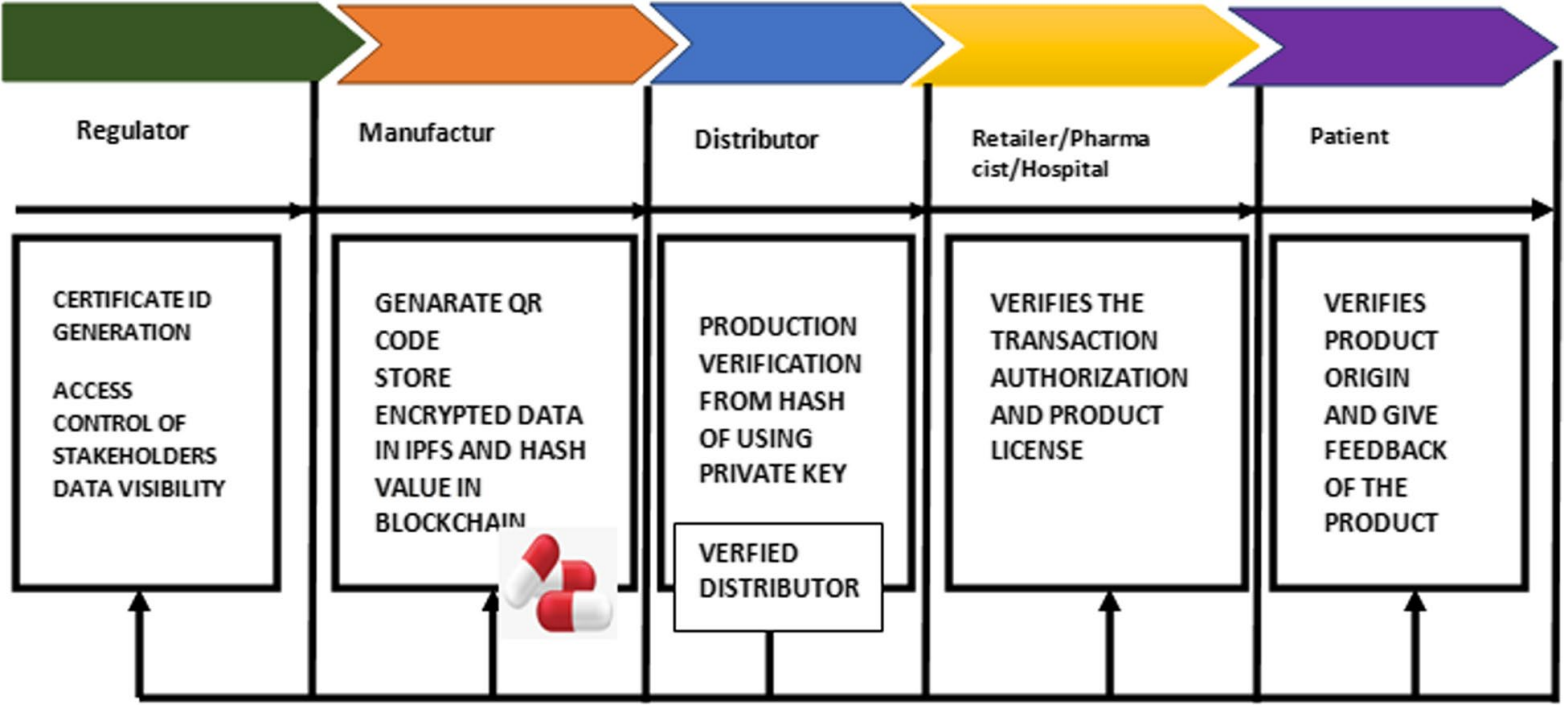
The Transaction Workflow

This step-by-step workflow, as shown in Fig. 6 is composed of four parts and five stakeholders.

#### Registration of companies

Registration is the first step for any entity with an interest in joining the supply chain network.

#### Registration of drugs

A manufacturing company must provide a ledger for recording all manufactured drugs with their license and QR code. Those data are encrypted and stored in the Cloud-based IPFS. The hash value of the product record is stored on the blockchain. Using the Cloud-based IPFS as part of the blockchain, the manufacturer provides transparency to supply chain stakeholders. The Cloud-based IPFS generates a hash ID once the transaction details are added, and then added to the blockchain. This can be used to track the transaction. A vehicle equipped with temperature sensors can be utilized to ship drugs to distributors via an IoT-enabled network. In IoT-enabled vehicles, stakeholders and government agencies can track the location of the vehicles in real-time, and thus track the delivery of drugs.

QR codes contain relevant information such as

• Date and time.
• The name of an item.
• Where it was manufactured and its expiration date.
• Location.

#### Hospitals/pharmacies receives drugs from distributors

After collecting medicine from logistics service providers, distributors can verify their origin using hash IDs stored on the blockchain. They can determine if it passed quality checks by tracking the information that the manufacturers added to the product. Pharma distributors are responsible for validating and signing off on the medicine received, which are subsequently added to the blockchain. The smart contracts are triggered by the signed transactions and the delivered drugs to hospitals, patients, and pharmacies.

#### The pharmacist receives the drugs and verifies their source

A hash ID saved on the blockchain allows pharmacists to trace the origin of drugs. False drug IDs may be sold to pharmacists and patients by a counterfeit drug distributor. Transactions that contain false information are invalid. Private keys provide protection against unauthorized transactions in the drug supply chain. Pharmacy personnel thus have the ability to spot anomalous transactions at the earliest possible stage. Pharmacists authorize transactions from distributors, adding them to the blockchain in order to ensure the sale is legal.

#### Patients purchase the drugs

The patients can verify that the medications are safe before they purchase them. Through their mobile app, they can check the source and quality standards of a drug by scanning the QR code attached to its packaging. They can also access the blockchain information through the QR code linked to the hash ID. By linking the blockchain ID of a drug to the patient's rating and feedback, patients can rate and comment on the drugs they purchase. Patients can rate a drug to help other individuals evaluate its effectiveness. Blockchain maintains immutable, transparent, and consensus-driven data on the drug supply chain. As the drug supply chain becomes more regulated, blockchain can help transform it into a surveillance network.

## Implementation of pharma supply chain system

The proposed framework has two parts in terms of the development environment. The separate backend and frontend development environments were used in this system. Docker is used to implement the backend functionality for a pharma supply chain management system. Implementation and experiments were carried out using a Core i7-8765u processor and 8 GB of memory as shown in Table 1. Docker was used for the Docker running environment, as well as for configuring images and containers, and Docker-compose was used for creating virtual machines. A project of the Linux Foundation, Hyperledger Fabric 1.4v [29], was used for our research. A Cloud-based IPFS is used as the off-chain database. The Fabric SDK requires Java and Node as prerequisites for client development. REST APIs make it possible to visualize backend business logic, such as user requests, assets, search, and transaction APIs. Frontend development was carried out using HTML5, CSS3 and JavaScript. To make our web application more efficient and user-friendly, we use third-party frameworks such as Query and Bootstrap. Frontend programming is done with a database, and backend programming is done with REST API servers. Clients use web applications to perform actions that trigger HTTP methods such as POST, GET, PUT, and DELETE, which then cause the web service to respond to HTTP responses according to the requests made by the client.

**Table 1:** Environment configuration

Component	Configuration
System under Test	Hyperledger Fabric 1.4v
CPU & Memory	Core i7-8765u and 8 GB
On-chain database	Couch DB
Off-chain database	Cloud based IPFS
Test language	Node.js,Java

### A business network’s building component

**Fig. 7:**
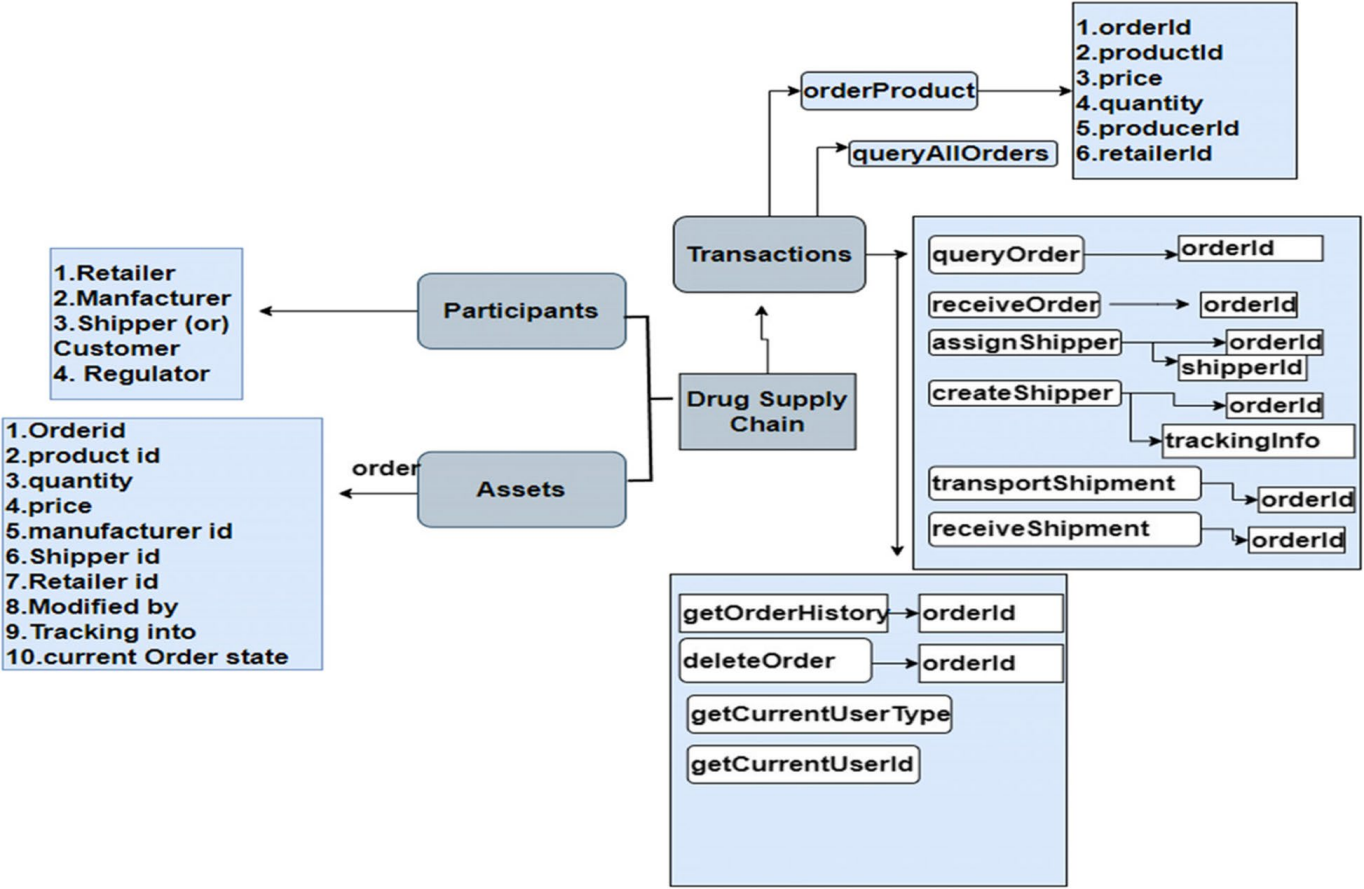
Business Network of the Proposed System

Participants, assets, and transactions are the three major components of a business network built on Hyperledger fabric. Production, shipping, retail, and the customer are all stakeholders in the network. In addition to the drugs, prescriptions, orders, and history of products, the assets include the repository. Figure 7 shows the transactions set functionality which is designed in our framework.

### Algorithm of the proposed system

Our proposed system is implemented with four user types: Customer, Shipper, Manufacturer, and Regulator [30]. New users must register through our admin interface. Once registered, users must enrol before logging in. There will only be one enrolment process as shown in Algorithm 1. Customers will be able to view the details and status of their orders using Algorithm 2 and the QR code. They must provide product ID, quantity, price, and producer ID to place an order. The producer will then receive the order. Manufacturers can view lists of their orders as well as their status. Each manufacturer has the option of accepting an order or rejecting it. The manufacturer must then assign a shipper to the order they have accepted. A shipper's details will be entered for the assignment. Products and their status can be seen by the shipper. As soon as the shipper accepts the shipment, it will be transported, and the status of the shipment will be changed from creation to transit. Once the shipment has been received by the retailer, the status will be changed to ‘delivered’. Each step will result in an update to the product status. When a customer enters a product ID, they can search for that particular item and access its details. Any product can be tracked by the regulator.

**Algorithm 1:**
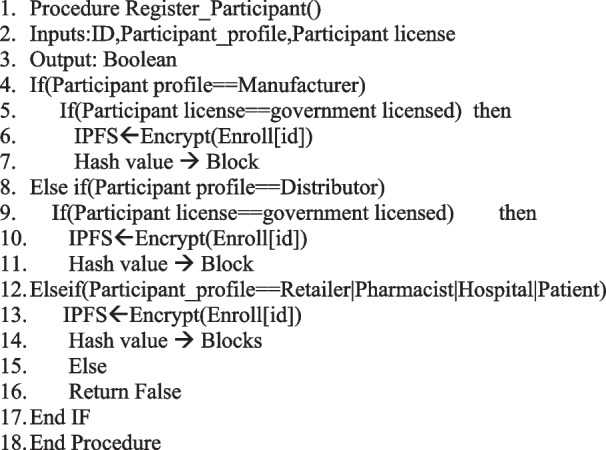
**Registration**

**Algorithm 2:**
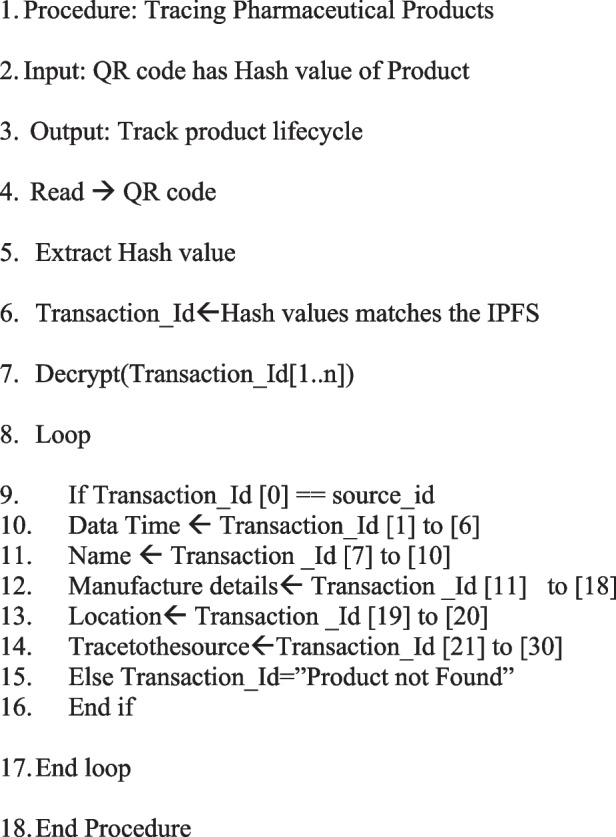
**Traceability**

**Algorithm 3:**
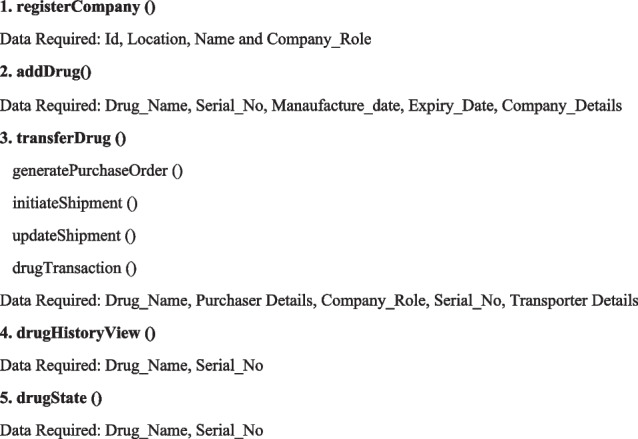
**Implemented Functionalities**

### Pharma supply chain-smart contract architecture

**Fig. 8:**
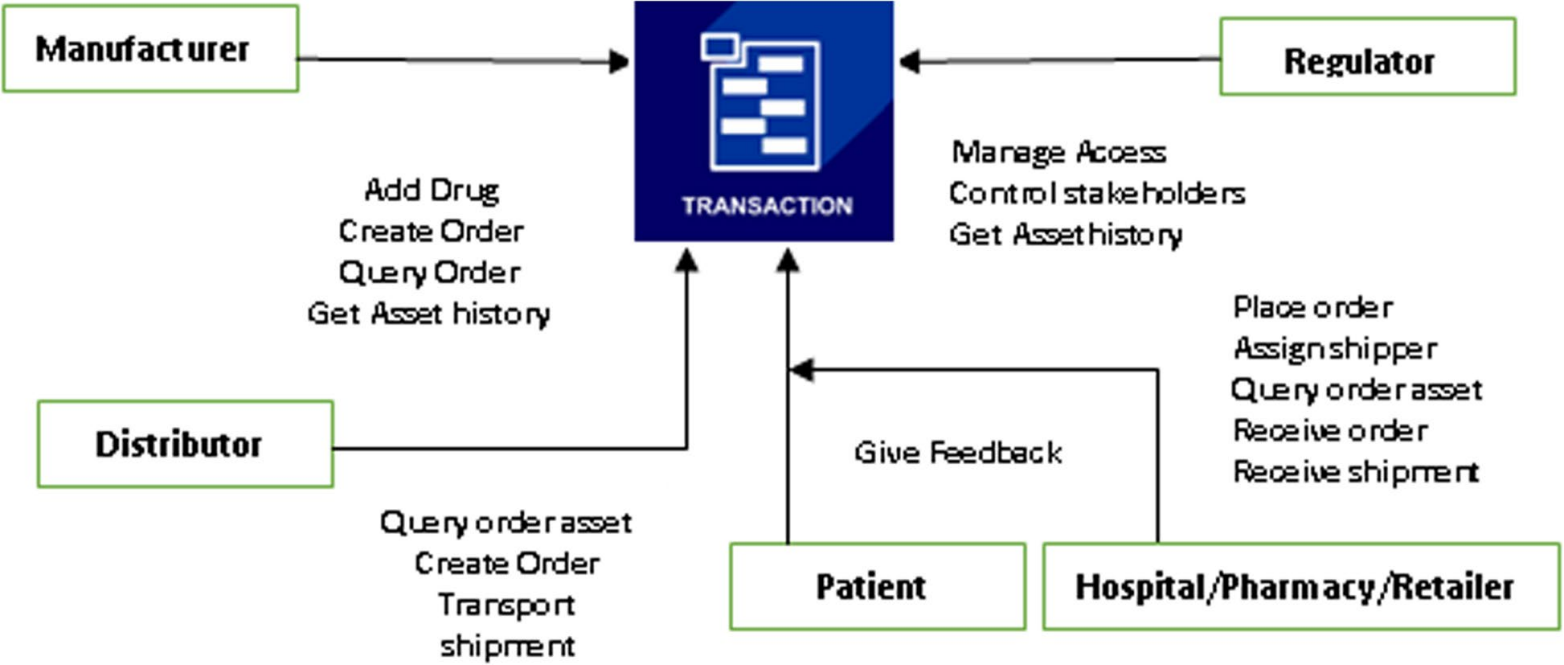
The Proposed Smart Contract Architecture

Chaincodes are written and deployed into the fabric for the stakeholders to ensure access control, privacy and transparency. For the proposed system, the three different modules of chaincodes are created (Fig. 8). They are:

• Registration of drugs by the provider
• Management of Assets
• Transaction update

### Pharma supply chain management in distributed ledger structure

A blockchain file system and a world state are the two core components of Hyperledger fabric's ledger technology. Databases that keep a cache of the ledger state's current state are known as world state databases. The world state contains key-value pairs that store the data. State data is stored in a key–value pair in the default state database used by smart contracts. A default database is embedded within the same process as an operating system at each node of the network. Smart contracts can be formatted into JSON documents using Couch DB. REST APIs are supported by Couch DB by enabling all kinds of requests to access data which can store all the transactions of the blockchain.

#### Implementation results

This proposed framework initially considers 1 organization which consists of peer 1, orderer and couch DB for implementation, and benchmarked using the system under a test. The Hyperledger fabric deployment has few steps as mentioned below:

- Start Hyperledger Fabric
- Initiate Smart Contract
- Create the Connection in the network
- Build and run the coded pharma supply chain applications
- Initiate the transactions

After the successful implementation, the assessment is made based on the following case studies.

##### Efficient drug transaction with attribute-based visibility

The framework used attribute-based access control that allows the visibility of product history based on the role of the stakeholders. The smart contract designed helps to monitor the visibility using a regulator. We can strengthen Quality Assurance and Pharmacovigilance by tracing products from the plant to the patient. Inventory visibility minimizes costs and waste. We can reduce revenue losses by identifying ‘grey trade’ incidents and strengthening brand trust.

##### Enhanced privacy and transparency

The framework is implemented in the permissioned Hyperledger fabric. The privacy and transparency of the supply chain is monitored using the Chaincode and distributed ledger which allows for the transparency of transactions to everyone in the network with attribute-based access control. The membership service providers maintain the identity of each stakeholder by providing the certificate using public key infrastructure.

##### Enhanced security

The technique of attribute-based access control allows the asset visibility to only allocated stakeholders. It has validated the Chaincode smart contract and certificate authority. Hence, information is confidential at each and every stage.

##### Enhanced scalability

These tests verify that our proposed system is able to handle large data with little increase in latency. This has been verified using the Hyperledger caliper benchmark.

##### Patient safety and quality assurance

It provides patients with information about the quality of their medications, and creates a feeling of security by making sure the medications are genuine and of high quality. It also facilitates the reporting of adverse events and recall of products more efficiently.

## Performance evaluation

Hyperledger Caliper was used to benchmark a blockchain-based application [31]. Caliper is designed to benchmark the performance of Hyperledger using different metrics such as throughput, latency, and success rate (average, minimum, maximum, and percentile). Furthermore, it indicates how resources such as CPU and memory will be allocated to the system. The result is calculated using the following metrics:

- Success and Failure rate
- Transaction/Read throughput
- Transaction/Read latency (minimum, maximum, average, percentile)
- Resource consumption (CPU, Memory, Network (Traffic in and Traffic out and Data read and write)

**Fig. 9:**
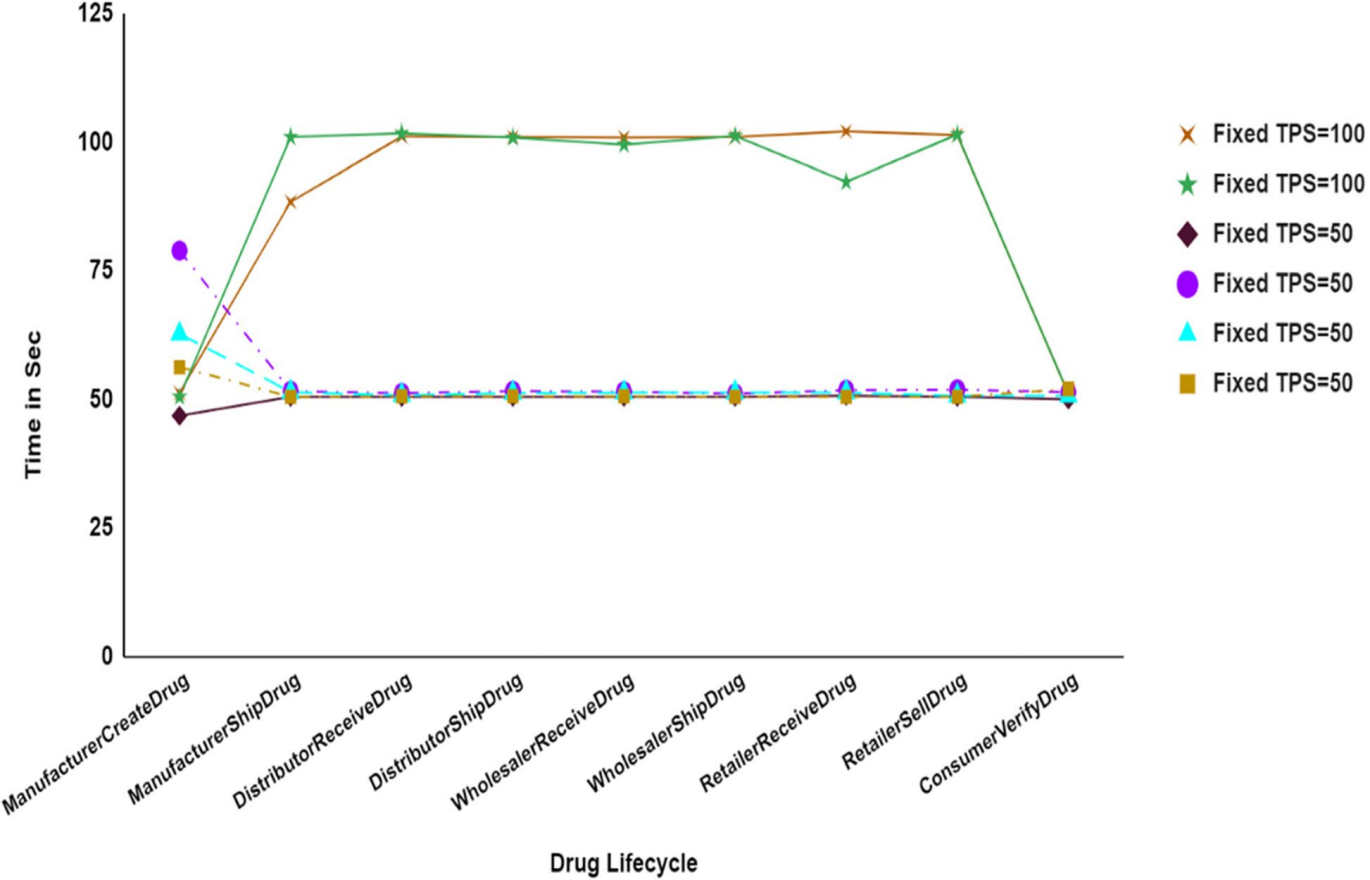
Comparative Analysis of Response Time with Different Fixed Transaction Sets

The system was initially tested for 1 organization and 1 peer node with various users such as Producer, Distributer, Retailer and Customer. The performance benchmarks are tested for manufacturerCreateDrug, manufacturerShipDrug, distributorReceiveDrug, distributorShipDrug, wholesalerReceiveDrug, wholesalerShipDrug, retailerReceiveDrug, retailerSellDrug, and consumerVerifyDrug. Figure 9 illustrates the comparison between two fixed transactions per second such as 50 and 100 for the consecutive set of 5 rounds of experiments with 100 users. Figure 11 shows that the response time increased when the fixed TPS in caliper increased from 50 to 100 during system under test. An analysis of the response time of the proposed system is provided in Fig. 12 by comparing three different user groups. A simulation was performed for a 100-ms period.

**Fig. 10:**
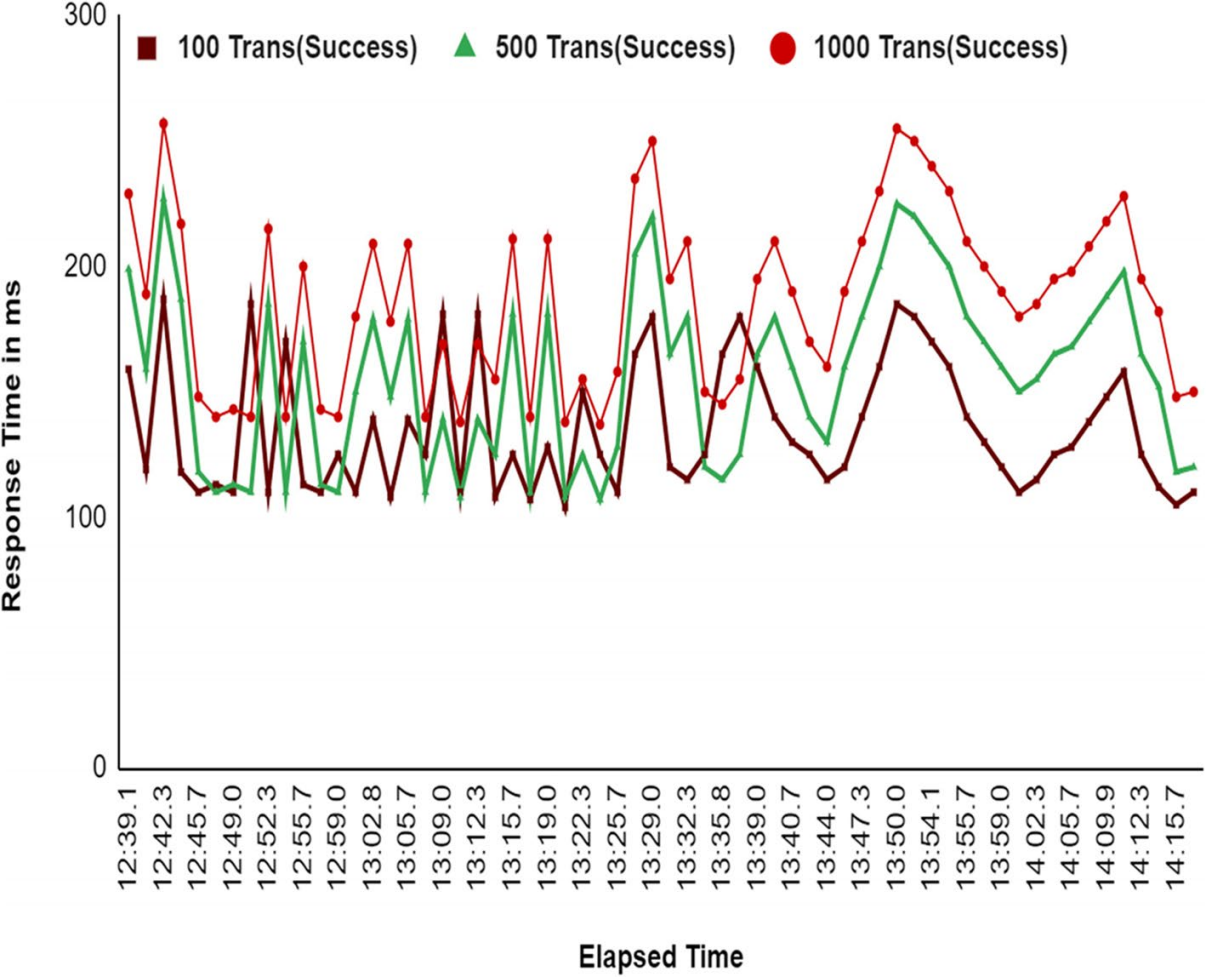
Comparative Analysis of Response Time with Three Different Sets of Transaction

We divided users into three groups based on transaction numbers: 100 transactions for round one, 500 transactions for round two, and 1000 transactions for round three. In Fig. 10, we see that the system responds almost the same way to the first two groups, but when we increase to 1000 transactions, the response time increases by only 30 ms. This means that even when the number of transactions increase, the system's response time stays the same.

**Fig. 11:**
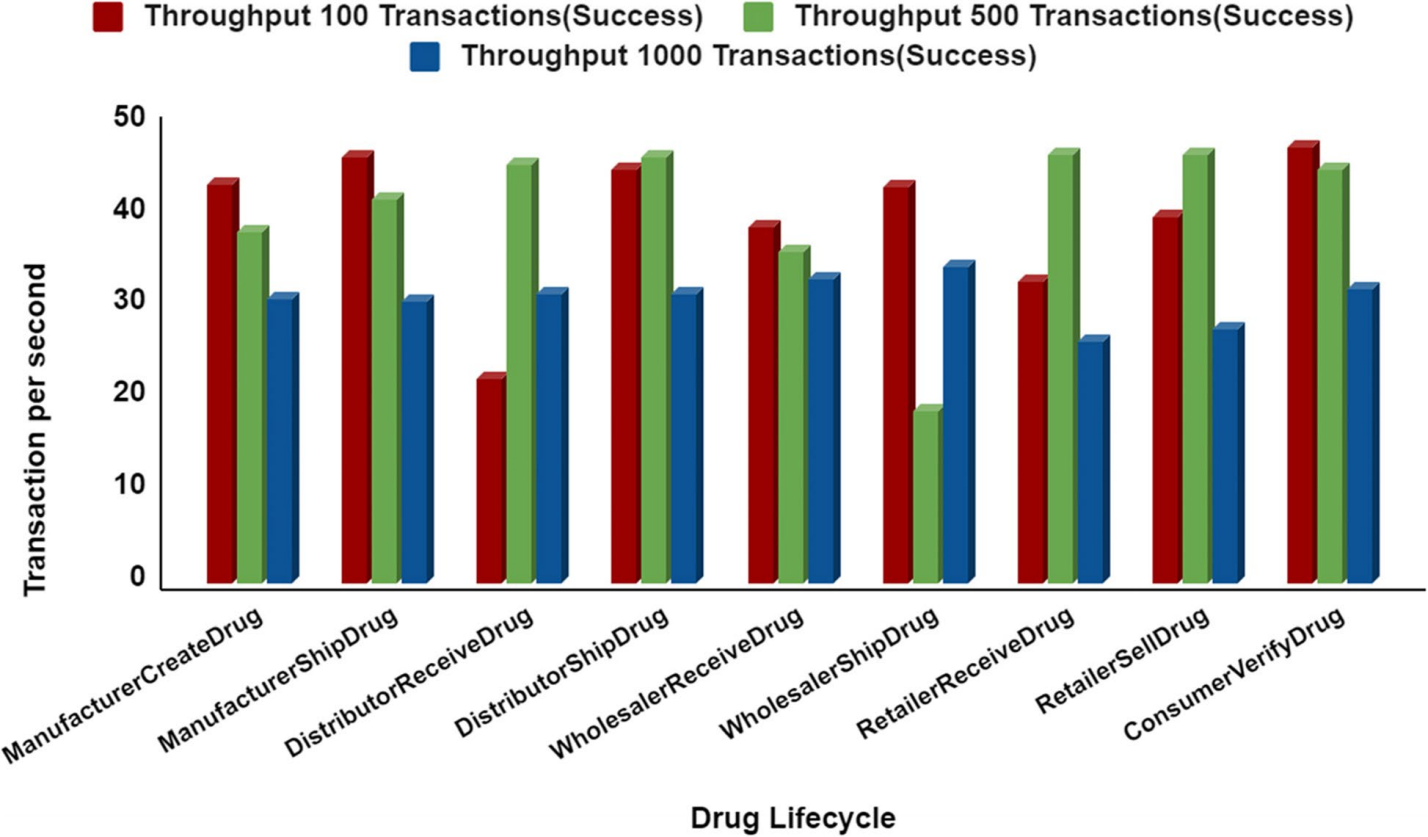
A Comparative Analysis of Throughput of Three Different Successful Transaction Sets

**Fig. 12:**
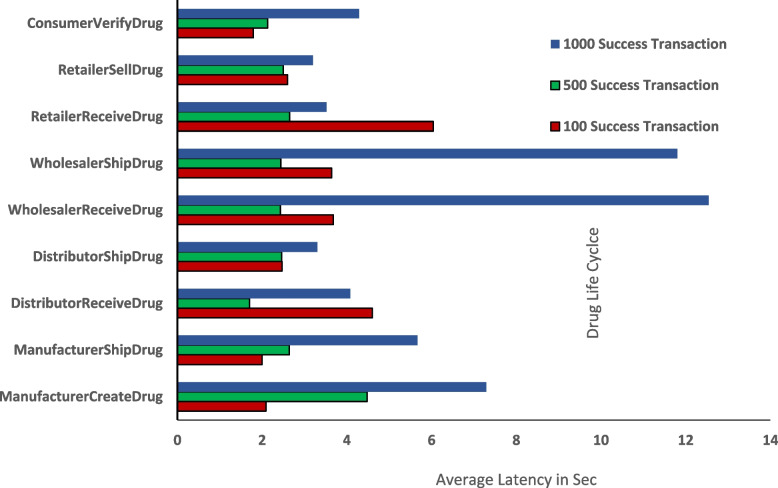
A Comparative Analysis of Average Latency of Three Successful Transaction Groups

In Fig. 11, we see that the number of transaction groups increase. The average successful transaction for 100 is 47, the average successful transaction for 500 is 46, and the average successful transaction for 1000 is 35. Hence, the average successful transaction has increased by only 25% when we increase the number of transactions from 100 to 1000. Figure 12 shows the average latency of 3 groups of successful transactions. For the 100 successful transactions, the average latency is 2.60 s. For the 500 successful transactions, the average latency is 3.21, and for the 1000 successful transactions, the average latency is 6.18. Thus, the average latency of successful transaction increases with increase in number of transaction group. Even though there is a little increase in latency, no failure or crash of network is recorded. Hence, the scalability of the proposed system is sustained when there is an increase in the number of transaction or users group.

Table 2 shows the average resource utilization of CPU, memory, and network of the proposed system. This reading has averaged from the 8 iterations of the proposed system. From the table, it is understood that the average resource utilization of a successful transaction is neither too high nor too low. Thus, our proposed system has efficiently utilized the system allocated resources.

**Table 2:** An analysis of the proposed system's resource utilization

Type	Name	CPU%(max)	CPU%(avg)	Memory(max) [MB]	Memory(avg) [MB]	Traffic In [MB]	Traffic Out [MB]
Docker	1OrgLocalFabric-Org1Peer1-drugsupplychainnet-0.0.1	107.65	18.76	149.56	142.25	10.06	11.90
	1OrgLocalFabric_orderer.example.com	41.60	5.97	126.08	102.51	0.06	27.18
	1OrgLocalFabric_ca.orderer.example.com	0.30	0.01	7.67	7.24	0.00	0.00
	1OrgLocalFabric_peer0.org1.example.com	83.41	22.04	65	62	43.19	34.78
	1OrgLocalFabric_couchdb0.org1.example.com	72.34	15.08	145	139.33	2.09	3.96
	1OrgLocalFabric_ca.org1.example.com	0.09	0.00	5.90	5.50	0.00	0.00

## Conclusion

Historically, traditional supply chains have been insecure, manual, anonymous, ineffective, opaque, centralized, and inaccessible. However, blockchain has proven its ability to transform the industry. Hacks and cyberattacks cannot occur on a blockchain. Blockchain technology is being used to digitize the drug supply chain, which will ensure security and transparency among users involved in it. To implement this proposed system, Hyperledger Fabric is considered as it is suitable for a permissioned network setup since the stakeholders will all have unique identifiers. Hyperledger fabric enables a user to have access controlled according to his or her role, therefore increasing the security and confidentiality of data. The system is reliable and always available since it is implemented in a distributed environment. The proposed system is transparent, secure, and private due to the use of smart contracts in conjunction with permissioned blockchain technology. Hence, drug trade is made more transparent and secure with blockchain technology. Utilizing blockchain technology, we have found that the proposed system achieves higher throughput, and lowers latency with minimal use of resources. Our current drug supply chain has loopholes that can be closed with the proposed model. In addition, Cloud-based blockchain frameworks can be used for scheduling real-time delivery, as well as detecting counterfeit drugs. There are a number of issues that can be addressed in future research, such as the development of a new supply chain framework for drugs that require extra handling. Blockchain technology is used in COVID-19 vaccine anti-counterfeiting frameworks.

## Data Availability

Data will be available based on the request.
